# A novel approach to sharing all available information from funded health research: the NIHR Journals Library

**DOI:** 10.1186/s12961-018-0339-4

**Published:** 2018-07-31

**Authors:** David Wright, Elaine Williams, Colin Bryce, Andrée le May, Ken Stein, Ruairidh Milne, Tom Walley

**Affiliations:** 1 National Institute for Health Research Evaluation, Trials and Studies Coordinating Centre, University of Southampton, Alpha House, Enterprise Road, Southampton, SO16 7NS United Kingdom; 2 Health Sciences, University of Southampton, University Road, Southampton, SO17 1BJ United Kingdom; 3 University of Exeter Medical School, St Luke’s Campus, Heavitree Road, Exeter, EX1 2LU United Kingdom; 4 Institute of Psychology, Health and Society, University of Liverpool, Liverpool, L69 3BX, United Kingdom

**Keywords:** Publishing, Information dissemination, Evidence-based practice

## Abstract

**Background:**

Relevant information on health research must be made publicly available in an accurate, timely and accessible manner if evidence is to inform practice and benefit patient care. Failure to publish research information represents a significant waste of research funds. However, recent studies have demonstrated that non-publication and selective or biased reporting remains a significant problem. The role of online publications in rectifying these issues by providing open access to study information is increasingly recognised.

**Objective:**

This paper details a novel approach to publishing research information developed by the National Institute for Health Research (NIHR), a major funder of health research in the United Kingdom. The NIHR has enhanced its Journals Library (www.journalslibrary.nihr.ac.uk), providing an online repository of information from research funded through five programmes. We describe how the NIHR Journals Library provides a ‘thread’ of relevant information for each study, including protocols, participant information sheets, data linkages, final reports, publications and diverse knowledge products. We also discuss the Library as a ‘living’ resource, one that is updated as each study progresses from inception to completion. Finally, we consider the implications of the Library for the NIHR, other journals and research teams submitting information.

**Conclusion:**

Openly publishing information from funded research in the NIHR Journals Library serves as a model of knowledge sharing, maximising return on investment and enhancing the usability and replicability of research findings for different evidence-user communities. The Library also supports wider ‘research on research’ ambitions, enabling users to interrogate the repository of NIHR-funded studies, enhancing the understanding of research commissioning, design, dissemination and impact.

Video abstract: www.youtube.com/watch?v=8H03uxN_iTE.

**Electronic supplementary material:**

The online version of this article (10.1186/s12961-018-0339-4) contains supplementary material, which is available to authorized users.

## Background

Research must be published accurately, in full and in a timely manner if it is to inform practice and benefit patient care. Failing to make evidence publicly available in a timely fashion represents a significant waste in research funds and is unethical by limiting impact on patient care [1–3]. Recent studies have shown that many studies remain unpublished years after completion and that a bias towards publishing studies with significant results remains [2–8]. These factors can lead to an over-estimation of treatment effect and restricts the availability of evidence to inform clinical practice and policy decision-making.

The applicability and replicability of published studies can be limited by incomplete or biased reporting [1, 9]. For example, descriptions of interventions in published trials often miss important information such as dose/duration of the procedure and control group comparators, a problem that is particularly prevalent in trials of complex interventions [10–12]. Comparisons of published trials with protocols or clinical study reports have revealed an underreporting of efficacy, adverse events and other patient-relevant outcomes in journal publications [13–15]. Published clinical trials may be selective in their reporting of analyses and outcomes, for example, favouring statistically significant results, and omitting or switching primary and secondary outcomes to obtain more favourable results [15–17]. Whilst reporting guidelines such as CONSORT, PRISMA and STARD have been designed and implemented to improve the quality and completeness of reporting, information on study design, analysis, interventions or outcomes often remains inadequate [18–20].

The development of online publications has begun to rectify these problems, providing opportunities for full open access of study information [21]. In 1999, Chalmers and Altman outlined how electronic resources could generate an online ‘thread’ of publications, starting with the protocol, continuing with reports from the resulting research and culminating with links to the complete dataset [22]. Online journals, such as *Trials*, have subsequently supported the ‘threaded’ concept by accepting a range of publications, including the protocol, first report of trial findings, secondary analyses, statistical analysis plans and lessons learned [18]. The Linked Reports of Clinical Trials project uses the CrossMark standard to include information about an article such as the clinical trial number and the trial registry, associating this information with the article DOI on publication. This means that queries on the CrossRef database identify articles related to the clinical trial number [23]. The OpenTrials initiative extends the Linked Reports of Clinical Trials approach further, establishing a database that includes not just publications but also other relevant trial information, including registry entries, sections of regulatory documents, structured data on methods, clinical study reports, blank consent forms and protocols [24]. Linking material such as statistical analysis plans, protocols and trial results in this manner has multiple benefits, maximising the availability of study information and enabling assessments of within-study reporting bias to be made.

Electronic publication also fundamentally challenges existing concepts of academic publishing, moving away from the production of a ‘final paper’ to establishing a single ‘living document source’, containing all relevant material that is updated as the study progresses [25]. Whilst the ambitions of living documents remain largely aspirational, they illustrate the potential of electronic publications [25].

The NIHR Health Technology Assessment (HTA) programme is a major funder of health research in response to NHS and health needs and has published full reports of the research it funds through its own peer reviewed, freely accessible, online NIHR journal (www.journalslibrary.nihr.ac.uk/hta) over the past 20 years. Researchers are obliged by contract to publish in the NIHR’s journal, which has yielded many benefits, including a 98% publication rate, excellent quality control and full accountability for the use of public money [26]. Open reporting through the journal supports the United Kingdom’s departments of health and social care’s continuing commitment to transparency in health research, and NIHR’s policy on open access. In 2013, the NIHR HTA publishing model was extended to four other programmes, namely *Efficacy and Mechanism Evaluation*, *Health Services and Delivery Research*, *Public Health Research* and *Programme Grants for Applied Research*, to collectively form the NIHR Journals Library (NJL; www.journalslibrary.nihr.ac.uk/).

The NJL is widely accessed, with over 230,000 unique visitors a year worldwide. Programmes fund various study designs and therefore the NJL covers a wide range of trials, evidence syntheses, observational studies and other methodologies. The NJL has recently evolved further, building on the concepts of threaded publications. The new NJL brings together, in one freely accessible site, all publications, documents and other web resources relating to NIHR-funded projects, as well as full reports when published in the online NIHR journals. The NJL supports living publications by establishing accounts of NIHR studies at their outset and developing these alongside each study. The new NJL is delivered as part of wider activity that seeks to maximise the transparency of funded research across the whole of the NIHR. This paper describes the information contained in the NJL and how the resource can be accessed by evidence users.

## Developing the archive

The concept of the living, threaded publication was developed from March 2015 through an iterative, participatory process involving NIHR Journals’ editors and advisory group members, patient and public contributors, researchers and research customers. We surveyed users of the NIHR Journals over a 4-month period, exploring views of the former NJL and identifying what new features participants would find most useful in the new site. Key requirements identified included access to scientific abstracts and plain English summaries, links to protocols, related project information (e.g. the commissioning brief to which the research was responding, or related systematic reviews), and other outputs from the same study.

## The NJL

**Fig. 1:**
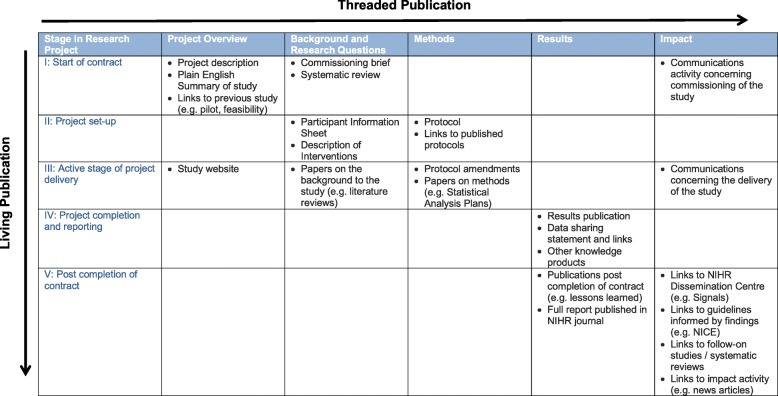
Overview of threaded, living publications provided in the NIHR Journals Library

The classic elements of a scientific paper have traditionally been the summary or abstract and the introduction (why the research was done?), the methods (how was it done?), the results (what was found?), and the discussion (what does it mean, strengths and weaknesses, and what next?). The NJL broadly reflects this successful and familiar structure. Figure 1 provides an overview of the information stored in the NJL.

**Fig. 2:**
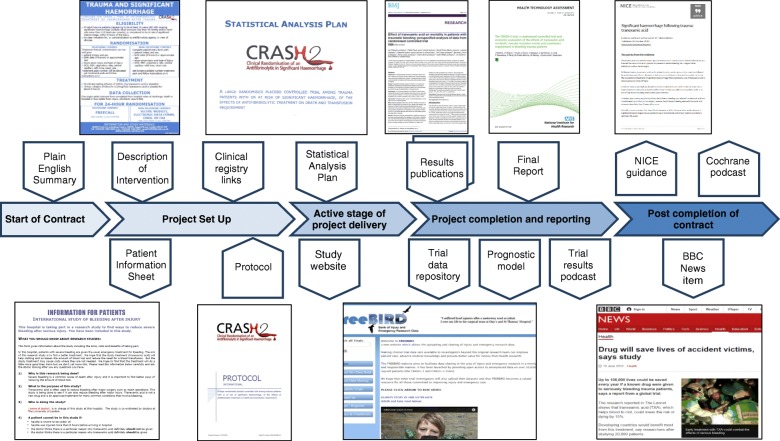
Thread of information provided for the CRASH2 trial in the NIHR Journals Library. The links in this figure can be seen in Additional file 1

Each study funded by the NIHR programmes has a unique project thread within the NJL. Figure 2 presents the types of information available for an NIHR HTA-funded study, the CRASH2 trial (a large randomised placebo controlled trial among trauma patients with significant haemorrhage of the effects of an antifibrinolytic treatment on death and transfusion requirement, www.journalslibrary.nihr.ac.uk/programmes/hta/0630320). The CRASH 2 study has been selected as it provides a particularly effective example of the richness and diversity of project information available through the NJL. Whilst not all projects will generate such a broad range of material, core information is available for all funded studies. Each project thread contains important details, including the chief investigator, cost, start and completion date, and any registry information.

The core of the thread on each project includes elements that are peer reviewed (e.g. background, methods, main results), and material provided by researchers that are not peer reviewed or are reviewed elsewhere, for which the library has no editorial responsibility (e.g. links to other journal publications). Links to relevant information are provided under five headings – project overview, background and research questions, methods, results and impact. Each heading will now be described with examples provided from the CRASH2 trial.

### Project overview (‘summary’)

This includes project descriptions extracted from applications, plain English summaries of the research and links to any previous studies associated with the project (e.g. prior systematic reviews, pilot and feasibility studies). Most information is provided as a requirement for NIHR-funded research. Links to the study’s own website are also provided, where they exist. The CRASH2 trial, for example, has its own website (www.crash2.lshtm.ac.uk) and links to a plain English summary of the study extracted from the application are provided.

### Background and research questions (‘introduction’)

This section describes the problem and sets out the research that has informed the question posed. It may include a commissioning brief or the researchers’ justification of the importance of the topic, supported by existing research, such as systematic reviews, feasibility or external pilots. All new research should undertake a prior systematic appraisal of the literature, ensuring it is informed by the best knowledge and does not duplicate what is already known [1]. This section of the NJL therefore makes such supporting evidence transparent. Links to relevant study documentation, such as participant information sheets and descriptions of interventions, are provided where appropriate. Links to relevant registries, such as ClinicalTrials.gov, are also provided. The CRASH2 trial project thread, for example, presents a Cochrane review that informed the study, links to the ClinicalTrials.gov registry and the United Kingdom Clinical Trials Gateway, and a Patient Information Sheet.

### Methods

Study protocols should be freely available as they reveal the initial intent of the research and facilitate quality assurance by enabling comparisons of original study outcomes with those ultimately reported [15, 27]. In recent years, the NJL has published study protocols and amendments with their justification and approvals, enabling studies to be reconstructed at each time point. An important element for trials is the statistical analysis plan, which is included as soon as it becomes available. In the case of evidence syntheses, appropriate statistical models used in complex meta-analyses and appropriate tools used for appraising study quality are included. The CRASH2 trial project thread, for example, includes a detailed protocol, a trial explanation, a statistical analysis plan and a description of intervention, in which eligibility criteria, randomisation and treatments are clearly set out.

### Results

Results develop over the course of a study. Early results may be no more than a record of recruitment rate or study newsletters. Results of sub-studies or methodological spin-offs may be available before the main study results. Links to the main results of a study are provided when available via links to peer-reviewed journals and to the full report when published in the NIHR programme’s peer reviewed, online journal. The full study report is a complete record of study findings, methods, literature informing the study, lessons learned and an update of the literature in light of the results. The CRASH2 trial, for example, has generated numerous outputs, including a full report and associated peer-reviewed articles, links to which are provided on the project thread. Links are also provided to a prognostic model and other knowledge products, including a podcast of trial results.

Providing access to study data is an important part of transparent reporting, supporting systematic reviews and meta-analyses, enabling further analysis on study data and informing the design of further research [28, 29]. Supporting data sharing from completed studies requires appropriate management including the protection of trial participants, maintained data standards and effective management of access and data repositories [30]. Researchers are often reticent in making data available even though data-sharing statements are now a requirement for many journals [31, 32]. NIHR-funded researchers have been obliged since 2014 to provide data sharing statements when submitting their final report and to share appropriately anonymised data if requested by the NIHR. Data sharing statements are made publicly available through the NJL. The CRASH2 trial, for example, has uploaded raw data from the study onto the Free Bank of Injury and Emergency Research Data (freeBIRD) repository, links to which are provided on the project thread. The NIHR is currently developing a practical policy to ensure that there is a clear route of managed access to study data, which will improve the usability and replicability of research.

### Impact

Authors may comment on and interpret their own results and ideally provide an updated systematic review that places the study in its research context, as well as a commentary on the implications of the results for patients, the NHS, health and social care. In some cases, studies will have proceeded unexpectedly and may not have been completed as planned. Accounts of such studies have an important place in the NJL as they may provide important lessons for other researchers and research funders. In all cases, there will be an opportunity for readers to respond to reports and to open a discussion.

The life of a project does not end with the publication of the final report, but extends to follow-on research, systematic reviews and meta-analyses, as well as uptake in clinical guidelines, other indicators of impact, or dissemination activities aiming to increase the likelihood of translation and uptake. The NJL showcases available evidence of impact from completed research, e.g. influence on organisations that provide guidelines and advice to improve health and social care in the United Kingdom, including the National Institute for Health and Care Excellence (NICE) or the National Screening Committee. The CRASH2 trial, for example, has generated a wide range of impact activity including NICE Guidance, a Cochrane podcast and a BBC News item, links to which are provided on the project thread.

#### How is the library populated?

Material in the NJL is derived from various sources. Parts of the NJL are populated from information created as part of the project funding process. All research commissioned and published in the NJL is managed through the NIHR programmes’ own research management systems, using a database that captures the research data from the point of application through to final publication. Research teams use the system to record key information, including the host institution, registry information, ORCiD iD, and publications in other journals relating to their NIHR-funded research. Peer review and editorial processes are also managed through these systems. Researchers are contractually obliged to notify NIHR programmes of relevant activities such as proposed changes to protocols, unexpected opportunities for methodological development and application and early study publications. Unlike other data repositories that rely on voluntarily submitted data from research teams or harvesting data from other websites, the NJL makes use of information already retained through effective research management. This efficiency results in low curation costs for NJL and makes the best use of information submitted by NIHR-funded researchers through contractual obligations.

Updated information on the database is extracted nightly, transformed and submitted to the NJL in XML format. Full reports published in the NIHR journals are typeset into an XML-first process during the production stage following copyediting. XML technology defines and structures report content through tagging, providing flexibility for journal content data by broadening the range of delivery formats. XML supports the storing, searching, managing and access of content, allowing the NJL to respond to users’ emerging needs. At the point of publication, the NJL uploads abstracts and copies of the final report to PubMed, PubMed Europe, NCBI Bookshelf and other indexing sites on behalf of the authors. The NJL is a member of CrossRef and each full report is assigned a DOI at publication stage.

#### Searching and exploring the library

The NJL homepage contains a search function allowing users to search for a condition/research area, a named researcher or other term. Once an area of interest has been selected, it is possible to refine the search using a range of criteria such as project status or Health Research Classification System health category. Selecting a specific project opens its own, unique project thread. Project threads are created at the point at which NIHR studies are contracted and are a ‘living’ resource in that each thread is populated as the project develops. Users can therefore return to specific projects and track through their development in an open, transparent and timely manner and receive alerts when a project is updated and when a final report is published in the relevant journal.

The NJL presents a ‘stacked’ view of study documents, enabling users to ‘drill down’ to the level of information required. Those wishing to access an overview of key findings can select the plain English summary on the project thread or access other knowledge products, such as conference presentations, uploaded to the Results section. Those requiring more information can retrieve the peer-reviewed, open access full report. People wishing to assess the quality and accuracy of reporting can download the study protocol and the study report, enabling comparisons to be made between initial study intentions and reported results. Those wishing to access the data to inform secondary analysis or meta-analysis can click on the data-sharing link in the results section of the project thread. Anyone wishing to review the outcomes from the research can click on the impact examples provided.

The NJL supports the wider ambitions of ‘research on research’ by providing open access to information on key stages in the funding and delivery of research. This supports the NIHR’s Research on Research programme as well as external academics and other users who can interrogate the NJL to understand, enhance and assess the quality of health research commissioning, design, dissemination and impact.

## Discussion, challenges and future ambitions

Developments in online publications have made it possible for relevant information on commissioned health research to be shared in an accurate, timely and accessible manner. The NIHR takes advantage of this and its distinctive position as a funder to push the boundaries of publication and make research more accessible to participants and users at every stage. The NJL presents a model that brings together the concepts of ‘threaded’ and ‘living’ publication, providing links to relevant information and materials relating to a specific study in a way that evolves over the life of each study. This obviates a problem often associated with living publications by retaining permanent versions of research through full reports [25].

The NJL develops the concept of threaded publication in other notable ways. Whilst much threaded-publication activity has, to date, focused on trial reporting [18, 23, 24], the NJL extends this to include information for all NIHR-funded studies, including evidence syntheses, qualitative studies and other designs. Furthermore, the NJL extends the information retained for each study beyond the completion of the research to include evidence of impact. Sharing impact information improves awareness of the implications of research and strengthens the understanding of what makes a project impactful, benefiting researchers, evidence-users and funders alike.

The NJL generates notable challenges. The creation of online journals has resulted in increased scrutiny of publication costs and publication options of traditional journals [33]. In recent years, there has been a rapid increase in the number of online open access journals [34]. Many journals seek to ensure exclusivity of reporting by tying authors into strict copyright arrangements. How such journals and authors respond to a living publication where some of the data may have been previously published and whether they will support links to the NJL remains to be seen. In the United Kingdom, the NJL may require new ways of capturing information in support of the Research Excellence Framework, an impact evaluation that assesses the research of British higher education institutions [35]. The Research Excellence Framework is informed heavily by assessments of research outputs, and the NJL thus presents new opportunities for impact assessment as publication threads may involve several outputs requiring appropriate weighting, and authorship may be more diffuse than previously reported.

Other challenges include assessing how ‘alive’ the NJL should be, since updating too frequently may risk the inclusion of inaccurate or misleading information. Furthermore, given that the uptake of findings after study completion may be diffuse and occur after many years, how should this information be collated in the long term?

We also need to ensure that the NJL is presented in ways that are transparent and understandable for all evidence-user communities, including patients and the public. Whilst the traditional publication format responds to academic interests, it is important to acknowledge that different evidence-user communities require diverse and appropriate knowledge products. These products may include the NIHR Dissemination Centre’s signals, webinars or tailored evidence summaries, often co-produced with the relevant user groups. We will support and monitor such diverse dissemination activities and products through the NJL. Finally, we acknowledge the potential overlap with related activity in the United Kingdom, including ResearchFish and OpenTrials, and with Higher Education Institutions’ systems such as PURE, and are making our information available to them. Supporting these enterprises ensures consistency of data entry and minimises the risk of duplicated effort for the researchers we fund.

We recognise that the NJL is taking a novel approach to sharing research information, necessitating ongoing quality assessment, monitoring and evaluation, and requiring further innovation and change over the coming years. We will share learning from the delivery of NJL to inform the reporting and dissemination activity of the wider NIHR and other funders and publishers. The success of the library will depend on effective partnerships between the research community, who will upload materials, and the NIHR as research funder and publisher. Some elements may prove unworkable, while others may be rendered obsolete by future developments, or simply lose value. However, it reflects NIHR’s determination to remain at the forefront of research and its dissemination and impact, and to continue to add value to research at every level.

## Conclusion

Transparent and timely access to relevant study information is essential if findings from health research are to be replicable and usable in practice. As a funder and publisher of research, the NIHR is able to ensure that all relevant information generated through the research it funds is publicly available. The relaunched NJL is an initiative that provides a ‘thread’ of relevant documents in a ‘living’ format. The resource requires new ways of working and information sharing with the researcher communities and it will take time for the NJL to reach its full potential. However, we anticipate that the NJL will serve as an effective model for sharing up-to-date, accurate information on all studies, maximising the return on investment and ensuring that the diverse interests of patients and the public, the scientific community, and those who plan and deliver health and social care services are effectively supported.

## Additional file

Additional file 1: Figure 2 with hyperlinks. (DOCX 1484 kb)

## Abbreviations

HTA: Health Technology Assessment
NICE: National Institute for Health and Care Excellence
NIHR: National Institute for Health Research
NJL: NIHR Journals Library
