# Supplementary material for: A novel approach to sharing all available information from funded health research: the NIHR Journals Library
Source: Health Res Policy Syst. 2018 Jul 31;16:70. doi: 10.1186/s12961-018-0339-4 (PMC6069813; doi:10.1186/s12961-018-0339-4)
Supplement: Supplementary file 1 — Figure 2 with hyperlinks. (DOCX 1484 kb) [file 12961_2018_339_MOESM1_ESM.docx]

[
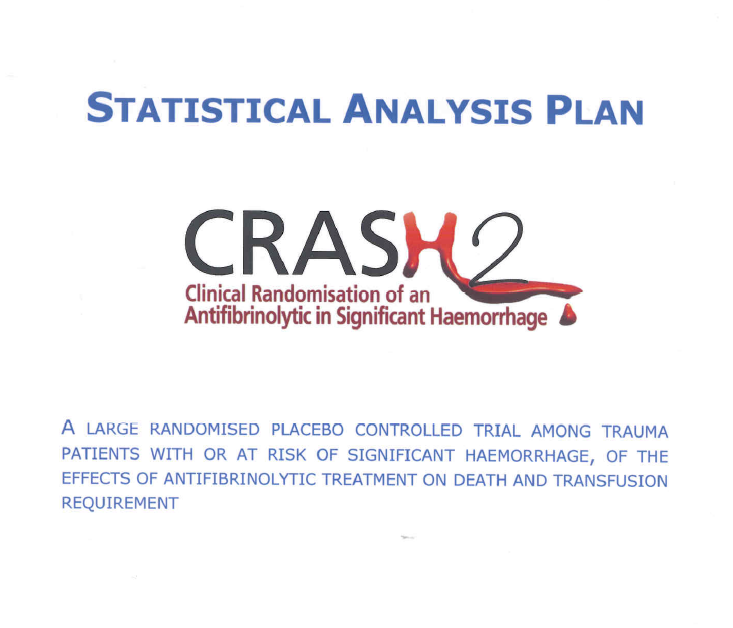
](https://njl-admin.nihr.ac.uk/document/download/2009609)[
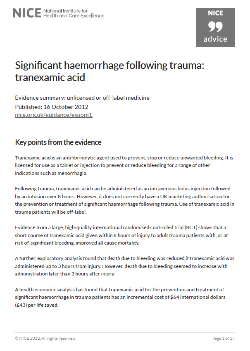
](http://www.nice.org.uk/advice/esuom1/ifp/chapter/about-this-information)[
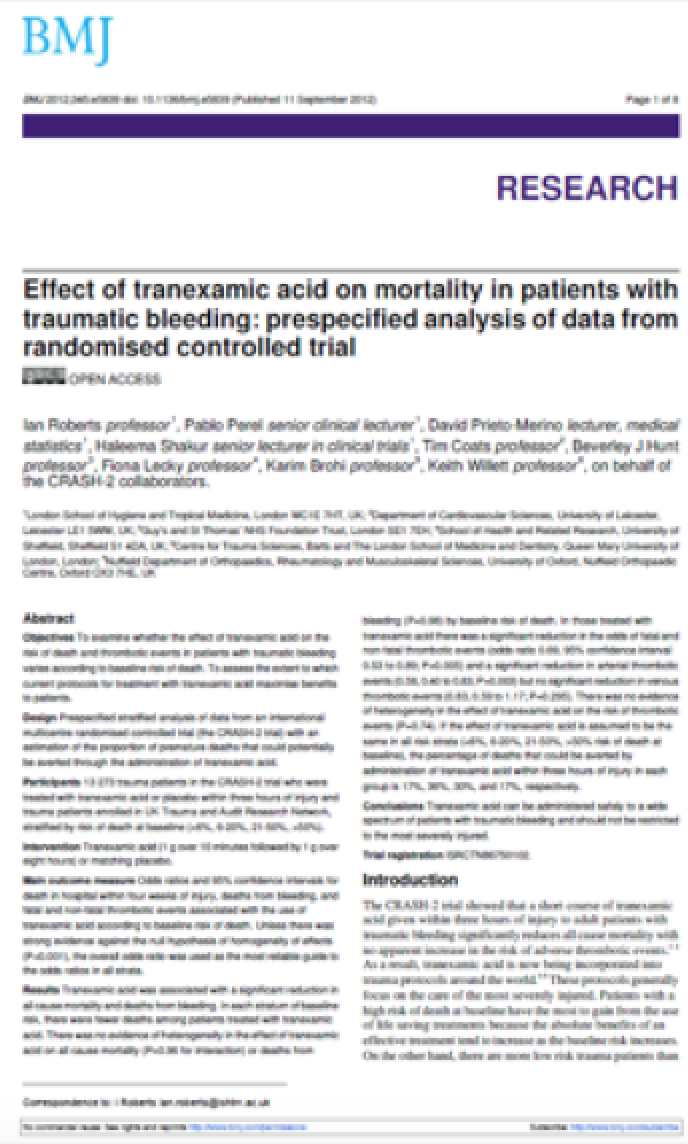
](http://www.bmj.com/content/bmj/345/bmj.e5839.full.pdf)[
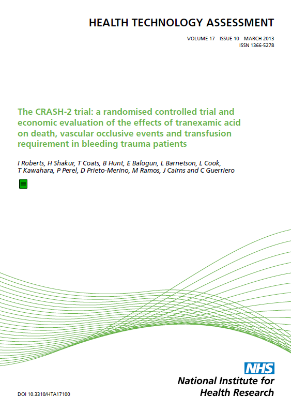
](https://njl-admin.nihr.ac.uk/document/download/2002365)[
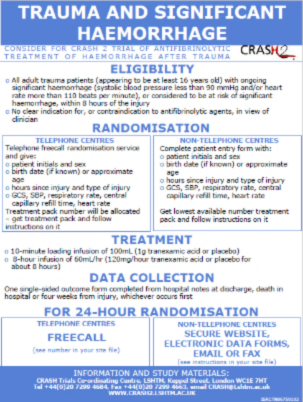
](https://njl-admin.nihr.ac.uk/document/download/2009606)[
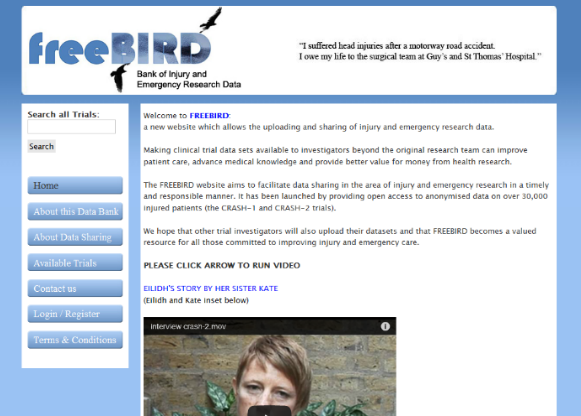
](http://ctu-app.lshtm.ac.uk/freebird/)[
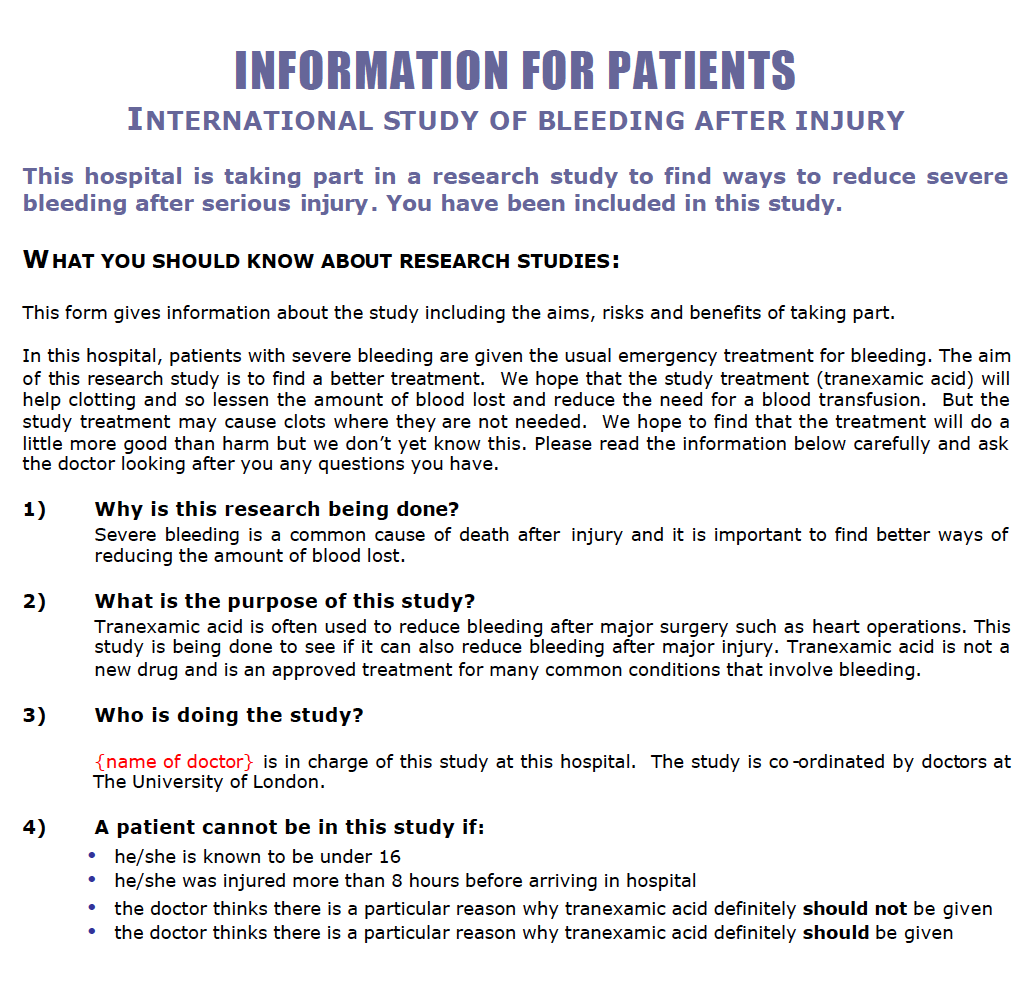
](https://njl-admin.nihr.ac.uk/document/download/2009607)

Trial

results

podcast

Prognostic

model

Trial

data

repository

Final

Report

**Start of Contract**

**Project completion and reporting**

**Active stage of**

**project delivery**

Study

website

Statistical

Analysis

Plan

Protocol

Patient

Information

Sheet

Clinical

registry

links

Description

of

Intervention

Plain

English

Summary

**Project Set Up**

Presentation of results

NICE Guidance

Final Report

Journal articles

Impact

articles

Models

NICE

guidance

Cochrane

podcast

Results

publications

**Post completion of**

**contract**

BBC

News

item

[
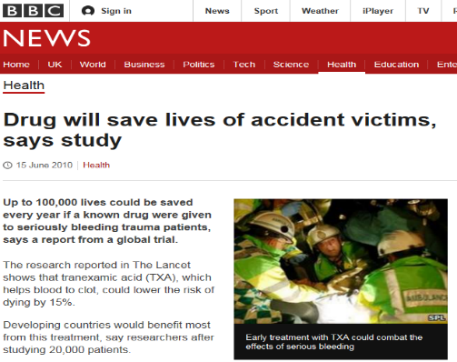
](http://www.bbc.co.uk/news/10311371)[
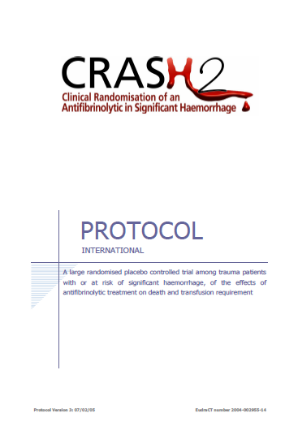
](https://njl-admin.nihr.ac.uk/document/download/2009463)

**Figure 1: Thread of information provided for the CRASH2 trial in the NIHR Journals Library**
